# Supplementary material for: The European Society of Gynaecological Oncology (ESGO), the International Society for the Study of Vulvovaginal Disease (ISSVD), the European College for the Study of Vulval Disease (ECSVD), and the European Federation for Colposcopy (EFC) consensus statement on the management of vaginal intraepithelial neoplasia
Source: Int J Gynecol Cancer. 2023 Mar 23;33(4):446–61. doi: 10.1136/ijgc-2022-004213 (PMC10086489; doi:10.1136/ijgc-2022-004213)
Supplement: Supplementary data [file ijgc-2022-004213supp001.pdf]

A systematic literature review of the studies published between January 2000 to April 2022 was carried out using the MEDLINE database. This search used indexing terms as follows: ablation, administration, adult, adverse effect, adverse event, aminolevulinic acid, aminoquinoline, anticarcinogenic agent, antineoplastic agent, antiviral drug, antiviral, brachytherapy, CO_2 laser vaporisation, cold knife, cold knife ablation, cold knife biopsy, complications, conservative surgery, conservative treatment, control, cytosine, DTC, estrogen therapy, excision, female, fertility, gynaecological surgery, gynaecological surgical procedure, gynecologic surgery, gynecologic surgical procedure, high grade vaginal intraepithelial lesion, human papillomavirus therapeutic vaccine, human, imiquimod, immune modulator, immune modulating drug, indole, irradiation, large loop excision, laser, laser ablation, laser method, laser therapy, loop electrosurgical excision procedure, loop electrosurgical excision procedure, loop electrosurgical excision procedure specimen, medical intervention, observation, organophosphonate, photochemotherapy, photodynamic therapy, photosensitizing agent, postoperative complication, postoperative recurrence, pre-invasive vaginal disease, quality of health care, quality of life, radiation therapy, radiofrequence ablation, radiofrequency, radiotherapy, recurrence, recurrent disease, relapse, reoperation, residual disease residual tumour, side effects, suction, surgery, surgical intervention, surgical management, surgical outcome, surgical outcome criteria, surgical procedure, surgical resection, surgical treatment, survival, survival rate, survival analysis, therapeutic agent, topical, vaccine, vaginal adenocarcinoma in situ, vaginal carcinoma in situ, vaginal dysplasia, vaginal intraepithelial neoplasia, vaginal melanoma, vaginal neoplasia, vaginal precancer.

The literature search was limited to publications in English, Italian, Spanish, Portuguese, German, and French. Priority was given to high-quality systematic reviews, meta-analyses, and randomised controlled trials but lower levels of evidence were also taken into consideration. Narrative reviews/guidelines and ongoing trials/protocols have also been collected (MEDLINE database, Cochrane Central Register of Controlled Trials, ISRCTN registry, ClinicalTrials (NIH), World Health Organization International Clinical Trials Registry Platform). The search strategy excluded editorials, case reports, letters, and in vitro studies.

A total of 97 articles were retrieved dealing with VaIN. Data extraction was performed for all the articles on treatment by two independent teams with double-check to ensure completeness. Tables with the most relevant clinical outcomes of 54 studies related to treatment of VaIN were completed and sumarized in the text.
